# Supplementary material for: Identification of photoperiod-induced specific miRNAs in the adrenal glands of Sunite sheep (Ovis aries)
Source: Front Vet Sci. 2022 Jul 22;9:888207. doi: 10.3389/fvets.2022.888207 (PMC9354845; doi:10.3389/fvets.2022.888207)
Supplement: Supplementary file 1 [file Data_Sheet_1.ZIP › Supplementary file/miRDeep2 file.zip!/miRDeep2 file/Know_Structure/SP42A2/oar-mir-487b.pdf]

miRBase precursor : oar-mir-487b
Total read count : 4713
oar-miR-487b-5p read count : 100
oar-miR-487b-3p read count : 4609
remaining reads : 4

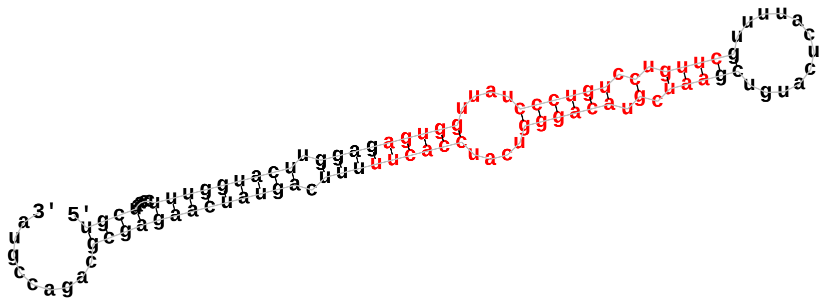

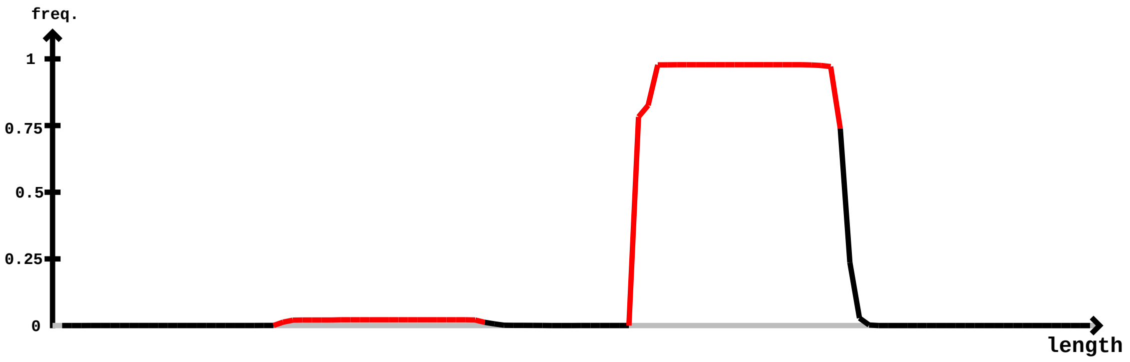

oar-miR-487b-3p

oar-miR-487b-5p

```
5'- ugcuaaccuuugguacuuggagagugguuaucccuguccuguucguuuuacucaugucgaaucguacagggucauccacuuuuucaguaucaagagcgcagaccgua   -3'   exp
    .(((....((((((((.((((((((....((((((.(.(((((............))))).).))))))....)))))))).)))))))))))..............   reads   mm   sample
    ..cuaaccuuugguacuuggag.....................................................................................   1       0    seq
    ....................agagugguuaucccuguccuguu................................................................   1       0    seq
    ......................agugguuaucccuguccugu.................................................................   4       0    seq
    ......................agugguuaucccuguccuguu................................................................   35      0    seq
    ......................agugguuaucccuguccuguuc...............................................................   16      0    seq
    ......................agugguuaucccuguccuguucg..............................................................   4       0    seq
    ......................agugguuaucccuguccuguucgu.............................................................   1       0    seq
    .......................gugguuaucccuguccuguu................................................................   5       0    seq
    .......................gugguuaucccuguccuguuc...............................................................   11      0    seq
    .......................gugguuaucccuguccuguucg..............................................................   17      0    seq
    .......................gugguuaucccuguccuguucgu.............................................................   1       0    seq
    ........................ugguuaucccuguccuguuc...............................................................   1       0    seq
    ........................ugguuaucccuguccuguucg..............................................................   2       0    seq
    ............................uaucccuguccuguucguuu...........................................................   1       0    seq
    ............................uaucccuguccuguucguuuu..........................................................   1       0    seq
    ............................uaucccuguccuguucguuuua.........................................................   2       0    seq
    .....................................................augucgaaucguacagggucauccacuu..........................   1       0    seq
    ...........................................................aaucguacagggucaucc..............................   5       0    seq
    ...........................................................aaucguacagggucaucca.............................   9       0    seq
    ...........................................................aaucguacagggucauccac............................   18      0    seq
    ...........................................................aaucguacagggucauccacu...........................   1068    0    seq
    ...........................................................aaucguacagggucauccacuu..........................   2246    0    seq
    ...........................................................aaucguacagggucauccacuuu.........................   339     0    seq
    ...........................................................aaucguacagggucauccacuuuu........................   2       0    seq
    ............................................................aucguacagggucauccacu...........................   16      0    seq
    ............................................................aucguacagggucauccacuu..........................   68      0    seq
    ............................................................aucguacagggucauccacuuu.........................   108     0    seq
    ............................................................aucguacagggucauccacuuuu........................   12      0    seq
    .............................................................ucguacagggucauccac............................   1       0    seq
    .............................................................ucguacagggucauccacu...........................   11      0    seq
    .............................................................ucguacagggucauccacuu..........................   51      0    seq
    .............................................................ucguacagggucauccacuuu.........................   534     0    seq
    .............................................................ucguacagggucauccacuuuu........................   108     0    seq
    .............................................................ucguacagggucauccacuuuuu.......................   10      0    seq
```

```
                                                                oar-miR-487b-3p
                      oar-miR-487b-5p
ugcuaaccuuugguacuuggagagugguuaucccuguccuguucguuuuacucaugucgaaucguacagggucauccacuuuuucaguaucaagagcgcagaccgua

..............................................................cguacagggucauccacuuu.........................    1           0           seq
...............................................................guacagggucauccacuu..........................    2           0           seq
```
